# Crystal structure of di-μ-chlorido-bis­[di­chlorido­bis­(methanol-κ*O*)iridium(III)] dihydrate: a surprisingly simple chlorido­iridium(III) dinuclear complex with methanol ligands

**DOI:** 10.1107/S2056989015006672

**Published:** 2015-04-22

**Authors:** Joseph S. Merola, Carla Slebodnick, Christopher Houser

**Affiliations:** a Department of Chemistry, Virginia Tech, Blacksburg, VA 24061, USA

**Keywords:** crystal structure, iridium, chlorido bridge, methanol ligand, hydrogen bonding

## Abstract

The reaction between IrCl_3_·*x*H_2_O in methanol led to the formation of small amounts of the title compound, [Ir_2_Cl_6_(CH_3_OH)_4_]·2H_2_O, which consists of two IrCl_4_O_2_ octa­hedra sharing an edge *via* chloride bridges. The mol­ecule lies across an inversion center. Each octa­hedron can be envisioned as being comprised of four chloride ligands in the equatorial plane with methanol ligands in the axial positions. A lattice water mol­ecule is strongly hydrogen-bonded to the coordinating methanol ligands and weak inter­actions with coordinating chloride ligands lead to the formation of a three-dimensional network. This is a surprising structure given that, while many reactions of iridium chloride hydrate are carried out in alcoholic solvents, especially methanol and ethanol, this is the first structure of a chloridoiridium compound with only methanol ligands.

## Abstract 2

While attempting to synthesize a cyclo­penta­dienyl iridium complex by the reaction between IrCl_3_·*x*H_2_O in methanol, several well-shaped crystals formed from the reaction mixture. Surprisingly, the crystals were of di-μ-chlorido-bis­[di­chlorido­bis­(methanol-κ*O*)iridium(III)] dihydrate, [Ir_2_Cl_6_(CH_3_OH)_4_]·2H_2_O. This is a surprising result in that, while many reactions of iridium chloride hydrate are carried out in alcoholic solvents, especially methanol and ethanol, this is the first structure of a chlorido-iridium compound with only methanol ligands.

## Chemical context   

The use of alcoholic solvents with IrCl_3_·*x*H_2_O for the formation of cyclo­penta­dienyl or olefin iridium complexes is exceedingly common (Herde *et al.*, 2007 ▸; Liu *et al.*, 2008 ▸, 2011 ▸; Morris *et al.*, 2014 ▸). Lately, we have been investigating the syntheses of half-sandwich iridium complexes with varying tetra­methyl­alkyl­cyclo­penta­dienyl ligands (Morris *et al.*, 2014 ▸). In all cases, the reaction takes place between IrCl_3_·*x*H_2_O and the tetra­methyl­alkyl­cyclo­penta­diene in methanol, either under thermal or microwave conditions. In most cases, the yields of of Cp*^R^ iridium chlorido-bridged dimers are good to excellent. Several reactions to synthesize Cp*^R^ iridium complexes with *R* = long-chain alkyls such as *n*-hexyl, *n*-heptyl and *n*-octyl produced good yields of the desired [Cp*^R^IrCl_2_]_2_ compounds but, in one instance, only produced a few crystals which turned out to be those of the title compound. Given the number of reactions that are carried out with IrCl_3_·*x*H_2_O in methanol, that this is the first time this compound has been seen by us or by any others active in the field is surprising.
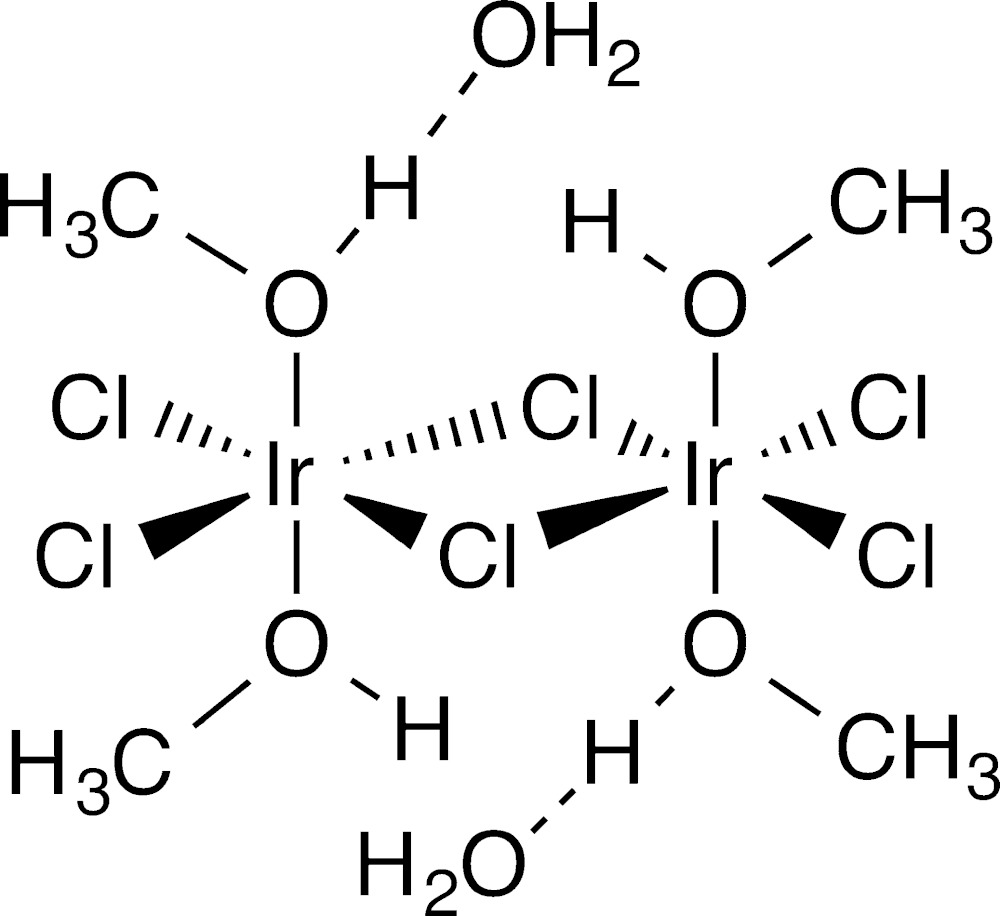


## Structural commentary   

**Figure 1:**
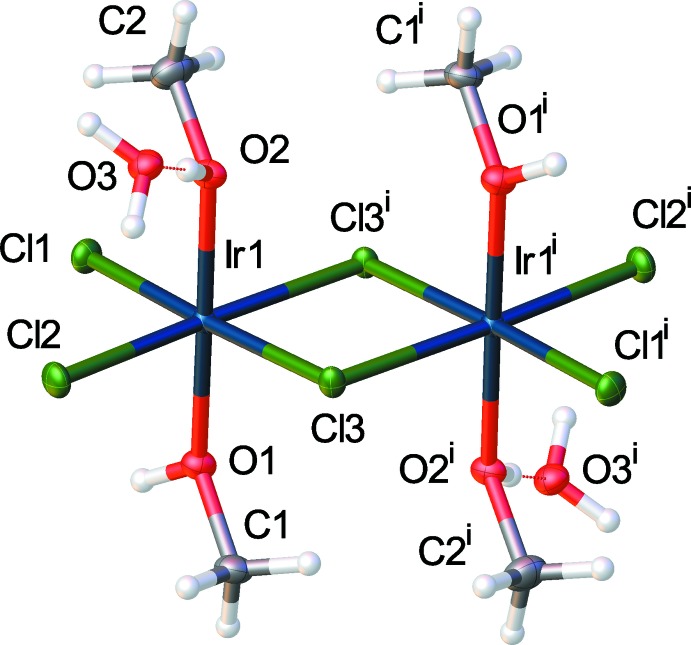
The full mol­ecular unit of the title compound with hydrogen-bonded lattice water mol­ecules [symmetry code (i) −*x* + 1, −*y* + 1, −*z* + 2]. Displacement ellipsoids are drawn at the 50% probability level.

The title structure (Fig. 1 ▸) consists of two iridium-centered octa­hedra sharing one edge *via* chloride bridges. For each octa­hedron, there are two terminal chloride ligands in the same plane as the bridging chloride ligands. The axial positions that complete the octa­hedra are occupied by O-bonded methanol ligands. One of the methanol ligands on each iridium atom is hydrogen-bonded to a lattice water. The two iridium-centered octa­hedra are related by an inversion center. The Ir—Cl bridges are symmetrical with identical Ir—Cl bond lengths of 2.385 (1) Å with two of the Ir—Cl bonds equivalent by symmetry and the unique bonds coincidentally equivalent [2.3847 (10) and 2.3846 (11) Å]. The only structure similar to the title compound currently in the Cambridge Structural Database (CSD version 5.35 with updates, Groom & Allen, 2014 ▸) is CCDC: CLESIR, bis­(μ-chlorido)­tetra­chlorido­tetra­kis­(di­ethyl­sulfide)­diiridium(III) (Williams *et al.*, 1980 ▸). The structural similarities between CLESIR and the title compound are that both contain octahedrally coordinated iridium atoms with the octa­hedra sharing one edge *via* Ir–Cl–Ir bridges. There are also two terminal chloride ligands on each iridium for both compounds. In the case of CLESIR, however, the remaining ligands on the iridium are di­ethyl­sulfido ligands. An additional difference is that, for the title compound, all chloride ligands are in the equatorial plane with methanol ligands occupying axial positions. For CLESIR, the di­ethyl­sulfido ligands on one iridium atom occupy axial positions but occupy equatorial positions on the second iridium.

## Supra­molecular features   

**Figure 2:**
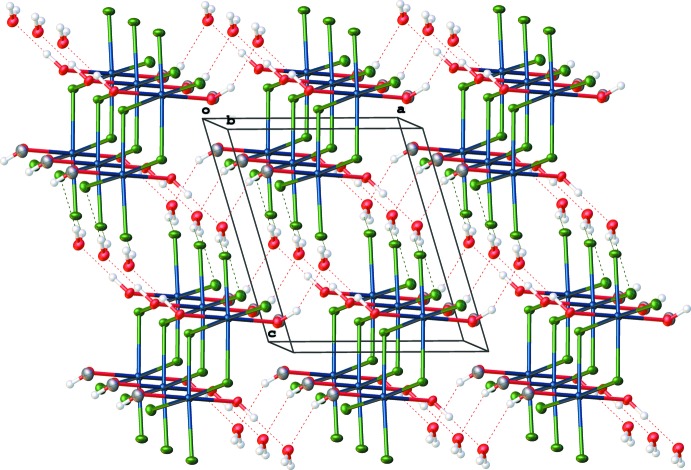
Packing diagram of the title compound showing the hydrogen-bonding (dashed lines) network. Displacement ellipsoids are drawn at the 50% probability level.

Each lattice water mol­ecule forms four hydrogen bonds linking four different iridium-centered dimers. Table 1 ▸ lists the various parameters describing the hydrogen bonding. As a donor, the water participates in two O—H⋯Cl bonds to chloride ligands on different mol­ecules while, as acceptor, the water participates in two O—H⋯O bonds to methanol oxygen atoms on two additional mol­ecules. A search of the CSD for O—H⋯Cl bonds between lattice water and chloride attached to any transition metal followed by analysis in *Mercury* (Macrae *et al.*, 2008 ▸) show that the O⋯Cl distances have a mean of 3.151 Å with a mean deviation of 0.055 Å. The two O⋯Cl distances of 3.208 (4) and 3.285 (3) Å for this structure places the distances at the high end of the range. However, when acting as acceptors, the lattice water displays O(methanol)—O(water) distances of 2.752 (5) and 2.647 (5) Å. A search of the CSD with analysis by *Mercury* (Macrae *et al.*, 2008 ▸) uncovers a mean O(donor)⋯O(acceptor) distance of 2.742 Å with a mean deviation of 0.085 Å, putting the donor–acceptor distances at the mean and slightly below the mean of these types of hydrogen bonds. Fig. 2 ▸ shows the hydrogen-bonding network that is created throughout the lattice of the title compound with the methanol methyl groups removed for clarity.

## Database survey   

A survey of the CSD found only 11 structures of iridium with methanol ligands (methoxide ligands were excluded from the search but they added only an additional eight structures to the result). Analysis with *Mercury* (Macrae *et al.*, 2008 ▸) found that Ir—O bonds in this small subset ranged from 2.185 to 2.317 Å with a mean of 2.251 Å and a standard deviation of 0.042 Å. The Ir—O bond lengths of the title compound of 2.066 (3) and 2.057 (3) Å are significantly smaller than the low end of this range. The small number of samples and the variety of structures available for comparison do not permit any clear conclusions as to the significance of these distances. All of the structures are of iridium(III) but the title compound is the only one with chloride as the sole other ligand set on each metal. While this structure determination was carried out at 100 K compared with room temperature for most of the other compounds with methanol ligands, such a significant bond shortening would not be expected based solely on temperature (Macchi & Sironi, 2004 ▸).

## Synthesis and crystallization   

IrCl_3_·*x*H_2_O and 1-heptyl, 2,3,4,5-tetra­methyl­cyclo­penta­diene were mixed in a round-bottom flask with 15 mL of MeOH and the reaction mixture was refluxed for two days. This procedure has been successfully used to synthesize a number of penta­alkyl­iridium chloride compounds in the past. After cooling to room temperature, the round-bottom flask was placed into a freezer overnight. There was no evidence of any product crystallization. The reaction mixture was then evaporated to dryness, yielding a tarry mixture. The tarry mixture was dissolved in diethyl ether and allowed to evaporate slowly. After the ether had evaporated, the mixture was again very tarry in appearance, but this time with a few crystals obvious in the flask. The structure of the title compound was determined from one of those crystals. It is unclear why this reaction did not proceed normally.

## Refinement   

Crystal data, data collection and structure refinement details are summarized in Table 2 ▸. H atoms bonded to C atoms were included in calculated positions with C—H = 0.96 Å and *U*_iso_(H) = 1.5*U*_eq_(C). H atoms bonded to water O atoms were included in calculated positions with O—H = 0.85 Å and *U*_iso_(H) = 1.5*U*_eq_(C). The H atoms bonded to methanol O atoms were refined independently with isotropic displacement parameters.

## Supplementary Material

Crystal structure: contains datablock(s) I. DOI: 10.1107/S2056989015006672/lh5757sup1.cif

Structure factors: contains datablock(s) I. DOI: 10.1107/S2056989015006672/lh5757Isup2.hkl

CCDC reference: 1057748

Additional supporting information:  crystallographic information; 3D view; checkCIF report

## Figures and Tables

**Table 1:** Hydrogen-bond geometry (, )

*D*H*A*	*D*H	H*A*	*D* *A*	*D*H*A*
O1H1O3^i^	0.86(1)	1.96(3)	2.752(5)	151(4)
O2H2O3	0.87(1)	1.78(1)	2.647(5)	179(2)
O3H3*A*Cl2^ii^	0.85	2.39	3.208(4)	160
O3H3*B*Cl1^iii^	0.85	2.45	3.285(3)	166

Symmetry codes: (i) 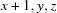; (ii) 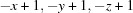; (iii) 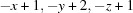.

**Table 2:** Experimental details

Crystal data	
Chemical formula	[Ir_2_Cl_6_(CH_4_O)_4_]2H_2_O
*M*_r_	380.68
Crystal system, space group	Triclinic, *P* 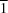
Temperature (K)	100
*a*, *b*, *c* ()	7.1445(4), 7.4876(5), 8.6362(7)
, , ()	73.597(6), 75.596(5), 89.404(5)
*V* (^3^)	428.37(5)
*Z*	2
Radiation type	Mo *K*
(mm^1^)	16.46
Crystal size (mm)	0.14 0.11 0.09

Data collection	
Diffractometer	Agilent Xcalibur Eos Gemini ultra
Absorption correction	Gaussian (*CrysAlis PRO*; Agilent, 2014 ▸)
*T*_min_, *T*_max_	0.184, 0.342
No. of measured, independent and observed [*I* > 2(*I*)] reflections	7987, 2817, 2575
*R*_int_	0.040
(sin /)_max_ (^1^)	0.750

Refinement	
*R*[*F* ^2^ > 2(*F* ^2^)], *wR*(*F* ^2^), *S*	0.028, 0.055, 1.03
No. of reflections	2817
No. of parameters	93
No. of restraints	6
H-atom treatment	H atoms treated by a mixture of independent and constrained refinement
_max_, _min_ (e ^3^)	1.67, 1.94

Computer programs: *CrysAlis PRO* (Agilent, 2014 ▸), *SHELXT* (Sheldrick, 2015*a* ▸), *SHELXL2014* (Sheldrick, 2015*b* ▸) and *OLEX2* (Dolomanov *et al.*, 2009 ▸).

## supplementary crystallographic information

### Crystal data

**Table:**

[Ir_2_Cl_6_(CH_4_O)_4_]·2H_2_O	*Z* = 2
*M**_r_* = 380.68	*F*(000) = 348
Triclinic, *P*1	*D*_x_ = 2.951 Mg m^−^^3^
*a* = 7.1445 (4) Å	Mo *K*α radiation, λ = 0.71073 Å
*b* = 7.4876 (5) Å	Cell parameters from 3445 reflections
*c* = 8.6362 (7) Å	θ = 4.0–32.1°
α = 73.597 (6)°	µ = 16.46 mm^−^^1^
β = 75.596 (5)°	*T* = 100 K
γ = 89.404 (5)°	Prism, clear light orange
*V* = 428.37 (5) Å^3^	0.14 × 0.11 × 0.09 mm

### Data collection

**Table:**

Agilent Xcalibur Eos Gemini ultra diffractometer	2817 independent reflections
Radiation source: Enhance (Mo) X-ray Source, Agilent	2575 reflections with *I* > 2σ(*I*)
Graphite monochromator	*R*_int_ = 0.040
Detector resolution: 16.0122 pixels mm^-1^	θ_max_ = 32.2°, θ_min_ = 4.0°
ω scans	*h* = −10→10
Absorption correction: gaussian (*CrysAlis PRO*; Agilent, 2014)	*k* = −11→11
*T*_min_ = 0.184, *T*_max_ = 0.342	*l* = −12→12
7987 measured reflections	

### Refinement

**Table:**

Refinement on *F*^2^	Primary atom site location: structure-invariant direct methods
Least-squares matrix: full	Hydrogen site location: mixed
*R*[*F*^2^ > 2σ(*F*^2^)] = 0.028	H atoms treated by a mixture of independent and constrained refinement
*wR*(*F*^2^) = 0.055	*w* = 1/[σ^2^(*F*_o_^2^) + (0.0194*P*)^2^] where *P* = (*F*_o_^2^ + 2*F*_c_^2^)/3
*S* = 1.03	(Δ/σ)_max_ = 0.001
2817 reflections	Δρ_max_ = 1.67 e Å^−^^3^
93 parameters	Δρ_min_ = −1.94 e Å^−^^3^
6 restraints	

### Special details

**Table:**

Geometry. All e.s.d.'s (except the e.s.d. in the dihedral angle between two l.s. planes) are estimated using the full covariance matrix. The cell e.s.d.'s are taken into account individually in the estimation of e.s.d.'s in distances, angles and torsion angles; correlations between e.s.d.'s in cell parameters are only used when they are defined by crystal symmetry. An approximate (isotropic) treatment of cell e.s.d.'s is used for estimating e.s.d.'s involving l.s. planes.

### Fractional atomic coordinates and isotropic or equivalent isotropic displacement parameters (Å^2^)

**Table:**

	*x*	*y*	*z*	*U*_iso_*/*U*_eq_	
Ir1	0.64307 (2)	0.65568 (2)	0.81418 (2)	0.01042 (5)	
Cl1	0.82335 (16)	0.93834 (15)	0.74853 (14)	0.0167 (2)	
Cl2	0.72146 (16)	0.65918 (17)	0.53518 (13)	0.0177 (2)	
Cl3	0.45411 (15)	0.36814 (14)	0.89543 (12)	0.01298 (19)	
O1	0.9022 (5)	0.5412 (5)	0.8390 (4)	0.0169 (7)	
H1	1.0118 (19)	0.603 (3)	0.785 (5)	0.025*	
O2	0.3880 (4)	0.7770 (5)	0.7908 (4)	0.0151 (6)	
H2	0.328 (5)	0.759 (4)	0.720 (4)	0.023*	
C1	0.9356 (7)	0.3485 (7)	0.8412 (6)	0.0208 (10)	
H1A	0.9287	0.3317	0.7366	0.031*	
H1B	0.8388	0.2669	0.9302	0.031*	
H1C	1.0615	0.3197	0.8584	0.031*	
C2	0.3605 (8)	0.9702 (7)	0.7877 (7)	0.0274 (12)	
H2A	0.4497	1.0503	0.6912	0.041*	
H2B	0.3835	0.9897	0.8868	0.041*	
H2C	0.2303	0.9984	0.7831	0.041*	
O3	0.2082 (5)	0.7157 (5)	0.5754 (4)	0.0182 (7)	
H3A	0.2516	0.6327	0.5284	0.027*	
H3B	0.2060	0.8178	0.5013	0.027*	

### Atomic displacement parameters (Å^2^)

**Table:**

	*U*^11^	*U*^22^	*U*^33^	*U*^12^	*U*^13^	*U*^23^
Ir1	0.01073 (8)	0.01068 (8)	0.00999 (8)	−0.00075 (6)	−0.00180 (6)	−0.00389 (6)
Cl1	0.0172 (5)	0.0126 (5)	0.0189 (5)	−0.0039 (4)	−0.0030 (4)	−0.0034 (4)
Cl2	0.0209 (5)	0.0215 (6)	0.0104 (5)	0.0007 (4)	−0.0018 (4)	−0.0058 (4)
Cl3	0.0167 (5)	0.0117 (5)	0.0113 (4)	−0.0021 (4)	−0.0022 (4)	−0.0056 (4)
O1	0.0149 (15)	0.0173 (17)	0.0199 (16)	0.0019 (13)	−0.0048 (13)	−0.0074 (13)
O2	0.0152 (15)	0.0185 (17)	0.0161 (15)	0.0035 (13)	−0.0073 (12)	−0.0092 (13)
C1	0.018 (2)	0.020 (2)	0.025 (2)	0.0057 (19)	−0.0036 (19)	−0.009 (2)
C2	0.028 (3)	0.017 (2)	0.036 (3)	0.003 (2)	−0.009 (2)	−0.006 (2)
O3	0.0196 (17)	0.0180 (17)	0.0184 (16)	0.0041 (14)	−0.0076 (13)	−0.0051 (14)

### Geometric parameters (Å, º)

**Table:**

Ir1—Cl1	2.3400 (11)	O2—C2	1.452 (6)
Ir1—Cl2	2.3267 (11)	C1—H1A	0.9600
Ir1—Cl3^i^	2.3847 (10)	C1—H1B	0.9600
Ir1—Cl3	2.3846 (11)	C1—H1C	0.9600
Ir1—O1	2.066 (3)	C2—H2A	0.9600
Ir1—O2	2.057 (3)	C2—H2B	0.9600
Cl3—Ir1^i^	2.3847 (10)	C2—H2C	0.9600
O1—H1	0.864 (10)	O3—H3A	0.8500
O1—C1	1.456 (6)	O3—H3B	0.8498
O2—H2	0.867 (9)		
			
Cl1—Ir1—Cl3^i^	92.90 (4)	C1—O1—H1	105.1 (16)
Cl1—Ir1—Cl3	177.08 (4)	Ir1—O2—H2	121.2 (16)
Cl2—Ir1—Cl1	91.47 (4)	C2—O2—Ir1	121.9 (3)
Cl2—Ir1—Cl3^i^	175.59 (4)	C2—O2—H2	104.7 (15)
Cl2—Ir1—Cl3	91.44 (4)	O1—C1—H1A	109.5
Cl3—Ir1—Cl3^i^	84.19 (4)	O1—C1—H1B	109.5
O1—Ir1—Cl1	83.45 (10)	O1—C1—H1C	109.5
O1—Ir1—Cl2	90.01 (9)	H1A—C1—H1B	109.5
O1—Ir1—Cl3^i^	91.08 (9)	H1A—C1—H1C	109.5
O1—Ir1—Cl3	96.79 (10)	H1B—C1—H1C	109.5
O2—Ir1—Cl1	94.91 (10)	O2—C2—H2A	109.5
O2—Ir1—Cl2	90.73 (9)	O2—C2—H2B	109.5
O2—Ir1—Cl3	84.81 (10)	O2—C2—H2C	109.5
O2—Ir1—Cl3^i^	88.30 (9)	H2A—C2—H2B	109.5
O2—Ir1—O1	178.22 (13)	H2A—C2—H2C	109.5
Ir1—Cl3—Ir1^i^	95.81 (4)	H2B—C2—H2C	109.5
Ir1—O1—H1	121.2 (16)	H3A—O3—H3B	109.5
C1—O1—Ir1	121.8 (3)		

Symmetry code: (i) −*x*+1, −*y*+1, −*z*+2.

### Hydrogen-bond geometry (Å, º)

**Table:**

*D*—H···*A*	*D*—H	H···*A*	*D*···*A*	*D*—H···*A*
O1—H1···O3^ii^	0.86 (1)	1.96 (3)	2.752 (5)	151 (4)
O2—H2···O3	0.87 (1)	1.78 (1)	2.647 (5)	179 (2)
O3—H3*A*···Cl2^iii^	0.85	2.39	3.208 (4)	160
O3—H3*B*···Cl1^iv^	0.85	2.45	3.285 (3)	166

Symmetry codes: (ii) *x*+1, *y*, *z*; (iii) −*x*+1, −*y*+1, −*z*+1; (iv) −*x*+1, −*y*+2, −*z*+1.
